# Compassion in healthcare: an updated scoping review of the literature

**DOI:** 10.1186/s12904-022-00942-3

**Published:** 2022-05-18

**Authors:** Sydney Malenfant, Priya Jaggi, K. Alix Hayden, Shane Sinclair

**Affiliations:** 1 Compassion Research Lab, University of Calgary, 2500 University Drive NW, Calgary, Alberta T2N 1N4 Canada; 2 Section of Palliative Care, Department of Family Medicine, Alberta Health Services, Zone, Calgary, Canada; 3 Faculty of Nursing, University of Calgary, 2500 University Drive NW, Calgary, Alberta T2N 1N4 Canada; 4 Libraries and Cultural Resources, University of Calgary, Calgary, Alberta Canada; 5 Division of Palliative Medicine Department of Oncology, Cumming School of Medicine, University of Calgary, 2500 University Drive NW, Calgary, Alberta T2N 1N4 Canada

**Keywords:** Compassion, Healthcare, Patients, Healthcare providers, Scoping review, Intervention

## Abstract

**Background:**

A previous review on compassion in healthcare (1988-2014) identified several empirical studies and their limitations. Given the large influx and the disparate nature of the topic within the healthcare literature over the past 5 years, the objective of this study was to provide an update to our original scoping review to provide a current and comprehensive map of the literature to guide future research and to identify gaps and limitations that remain unaddressed.

**Methods:**

Eight electronic databases along with the grey literature were searched to identify empirical studies published between 2015 and 2020. Of focus were studies that aimed to explore compassion within the clinical setting, or interventions or educational programs for improving compassion, sampling clinicians and/or patient populations. Following title and abstract review, two reviewers independently screened full-text articles, and performed data extraction. Utilizing a narrative synthesis approach, data were mapped onto the categories, themes, and subthemes that were identified in the original review. Newly identified categories were discussed among the team until consensus was achieved.

**Results:**

Of the 14,166 number of records identified, 5263 remained after removal of duplicates, and 50 articles were included in the final review. Studies were predominantly conducted in the UK and were qualitative in design. In contrast to the original review, a larger number of studies sampled solely patients (*n* = 12), and the remainder focused on clinicians (*n* = 27) or a mix of clinicians and other (e.g. patients and/or family members) (*n* = 11). Forty-six studies explored perspectives on the nature of compassion or compassionate behaviours, traversing six themes: nature of compassion, development of compassion, interpersonal factors related to compassion, action and practical compassion, barriers and enablers of compassion, and outcomes of compassion. Four studies reported on the category of educational or clinical interventions, a notable decrease compared to the 10 studies identified in the original review.

**Conclusions:**

Since the original scoping review on compassion in healthcare, while a greater number of studies incorporated patient perspectives, clinical or educational interventions appeared to be limited. More efficacious and evidence-based interventions or training programs tailored towards improving compassion for patients in healthcare is required.

**Supplementary Information:**

The online version contains supplementary material available at 10.1186/s12904-022-00942-3.

## Background

Compassion in healthcare has continued to receive growing interest over the past decade [1] from researchers, educators, clinicians, policy makers, patients, and families alike, with patients strongly emphasizing its importance to their overall quality of care [2–5]. Compassion has been associated with a positive impact on the patient experience and a variety of patient-reported outcomes – specifically, reduced patient symptom burden [6–8], improved quality of life [6, 9–11], and even an enhancement in quality-of-care ratings [5, 6, 12–16]. While compassion is recognized as a standard of care and a core component of patients’ healthcare experience, it is also been found to be lacking in terms of its provision [3, 5, 6, 12, 17–23] and in much need for improvement [24–28]. A lack of compassion has been associated to increased patient/family complaints, healthcare costs, and adverse medical events [19, 24, 29–36]. Both the Canadian and American Medical Associations list compassion as one of their core virtues exemplified by the ethical physician [37, 38], with the Canadian Medical Association (CMA) stating that “a compassionate physician recognizes suffering and vulnerability, seeks to understand the unique circumstances of each patient, attempts to alleviate the patient’s suffering, and accompanies the suffering and vulnerable patient” (p.2) [37]. Furthermore, researchers agree that while compassion is vital across healthcare settings, it is a central goal and tenant of quality palliative care where multifactorial suffering is prevalent, requiring future research, including how it can be sustained in palliative care providers [39–42]. However, it was only recently that compassion was delineated from a related construct, empathy (i.e. the ability to resonate with another’s positive or negative feelings) [43, 44], highlighting *action* as one of its additional, yet paramount components [6, 44, 45]. The necessity of action within conceptualizations of compassion was independently affirmed by both palliative care patients' and palliative care providers' definitions of compassion, with patients defining compassion as “*a virtuous response that seeks to address the suffering and needs of a person through relational understanding and action*” [6], and healthcare providers (HCPs) defining compassion as: *“a virtuous and intentional response to know a person, discern their needs and ameliorate their suffering through relational understanding and action”* [45]. With the emergence of empirical models and definitions of compassion [6, 45], and a valid and reliable patient-reported compassion measure for research and clinical use [15], studies have now shifted towards determining whether compassion can be trained or cultivated in practicing clinicians, while nurturing and sustaining the innate qualities related to compassion that these individuals already possess [8, 46–49]. Recent studies suggest that while compassion is largely inherent, it can be influenced by life experiences and can fluctuate over time [50, 51]. Educational leaders within healthcare settings have also emphasized the need to incorporate compassion training into their curriculum [52]. A systematic review found that implementing various curriculum strategies could result in practicing clinicians enhancing their overall levels of compassion and empathy, as perceived by the physician participants themselves, patients, standardized patients, or third-party observers, using a variety of measurement tools [52]. While recent reviews have identified the current landscape of compassion training programs [53, 54], (i.e. those with the goal of cultivating compassion in others), studies identified in these reviews still present significant limitations such as: an absence of training to develop HCP skills within the interpersonal domains of compassion; lack of multi-modal training programs for practicing HCPs; reliance on self-reported assessments of learning outcomes as opposed to patient-reported outcomes; and a lack of Randomized Controlled Trials (RCTs) and longitudinal studies determining the retention and integration of skills into clinical practice [54]. As such, developing compassion training that is empirically based, clinically relevant and addresses these limitations is required and necessary in evidence-based, patient-centred healthcare delivery [54].

Despite remarkable efforts towards enhancing compassion in healthcare and a burgeoning knowledge base on the topic, the academic literature on compassion in healthcare remains deficient, specifically in regard to how compassion is perceived by patients themselves – the ultimate beneficiaries [1, 55]. This lack of patient perspectives was a key limitation identified in a previous scoping review by Sinclair et al. (2016), a study which undertook a synthesis of the existing literature within a 25-year period (1988-2014) in order to determine what is known about compassion in healthcare. This original scoping review demonstrated an array of study types, settings, participant types (i.e. clinicians and patients), operational definitions and cultivation techniques, while also affirming the interpersonal nature of compassion, its predication on action, and associated barriers and facilitators in both education and practice [1]. Interestingly, patients themselves were widely underrepresented throughout the identified studies, with only 30% of them including patients, largely in a limited fashion, and the remainder focusing on clinicians, and/or students, and/or caregivers. Studies also failed to include patient-derived definitions of compassion, and studies that exclusively sampled patients and/or outcomes related to patients’ health and quality of life were also lacking [1]. Further, of the compassion interventions that were identified in this original review, only two were randomized controlled trials evaluating clinical interventions, and eight were educational interventions, of which only two of the interventions used validated tools to measure compassion – one of which used a tool measuring empathy [1]. The absence of a comprehensive knowledge base and an ambiguous understanding of how compassion is conceptualized by patients and HCPs in various healthcare contexts, makes operationalizing and improving its delivery to patients an extremely daunting and challenging task.

**Fig. 1:**
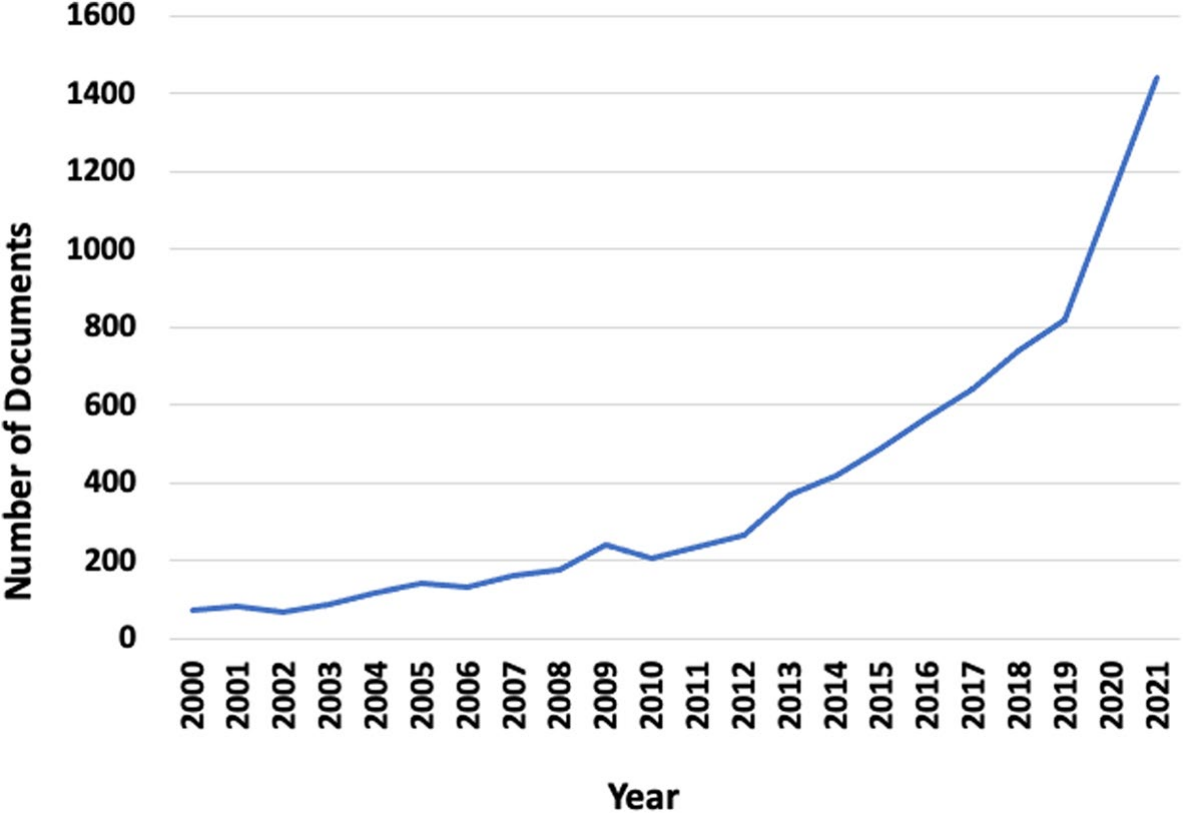
Number of documents from 2000 to 2021 (Scopus search: Compassion in healthcare)

Despite considerable advancement in the field of compassion in healthcare over the past 30 years, including the identification of associated research gaps and recommendations to guide research [1], there has been a rapid influx of disparate studies over the past 5 years (Fig. 1) that require a further mapping of the literature to determine if previously identified limitations have been addressed and if any new domains of compassion research have emerged. Therefore, in keeping with the iterative nature of scoping reviews [56, 57], the objective of this scoping review was to provide an update to our original review [1] to include contributions to the healthcare literature over the past 5 years. The review question was: What is currently known about compassion in healthcare? In addition to an overview of how the field of compassion in healthcare has evolved, readers of this review will gain evidence-based knowledge in four specific areas: 1) the nature of compassion and how it is conceptualized in the healthcare literature; 2) the feasibility and reputed impact of clinical and educational compassion interventions; 3) challenges and enablers to integrating compassion in contemporary healthcare; and 4) whether compassion can be meaningfully and rigorously measured.

## Methods

### Search strategy

Through consultation with a research librarian (KAH), a search for existing knowledge synthesis reviews on compassion in healthcare was performed using MEDLINE, Google Scholar, and Prospero. Apart from the original scoping review [1] and a more recent review targeting compassion in the pediatric population [58], no other completed knowledge synthesis reviews were identified on this topic.

The current scoping review is an update of the original scoping review published in 2016 [1]. A study protocol was written a priori to guide this current review, which is reported in accordance with the PRISMA-ScR reporting guidelines [59]. The included studies from the original review (*n* = 44) were first analyzed for keywords and subject headings by KAH. The search in the original review was intentionally broad and included terms such as “delivery of healthcare, healthcare, palliative, palliative care, end-of-life, terminal, end-of-life care, terminal care, terminally ill patient, euthanasia, cancer, neoplasm, carcinoma, tumor, religion, spirituality” (p.2) [1]. These terms, however, did not show up consistently in the 44 included studies of the original review and as such, were not included into the refined updated search strategy. This initial analysis determined that two concepts were constant across the 44 studies: compassion and HCPs, becoming the focus of the refined search for the current review. For each concept, both keywords and subject headings were utilized, where keywords were the same for all databases, and subject headings were defined by each database’s controlled vocabulary. The draft search strategies were tested to ensure all the original included studies were captured. Once the search strategy was finalized, it was limited to English and date limits of January 1, 2015 – November 2020. The original review included studies published up to December 31, 2014. The final searches were run between November 16 and November 27, 2020, and results uploaded into Covidence, with each upload automatically deduplicated.

Congruent with our original review, the following databases were searched: MEDLINE(R) and Epub Ahead of Print, In-Process & Other Non-Indexed Citations and Daily 1946 to November 16, 2020 (OVID), Embase 1974 to 2020 November 25 (OVID), EBM Reviews - Cochrane Central Register of Controlled Trials October 2020 (OVID), APA PsycInfo 1806 to November Week 32,020 (OVID), CINAHL Plus with Full Text (Ebsco), Academic Search Complete (Ebsco), and Scopus (Elsevier). Additional file 1 provides the search strategies for each database.

### Eligibility criteria

Studies that sampled inpatients or outpatients, and/or qualified clinicians (e.g. physicians, nurses, healthcare aides) were included in the final analysis. Studies were excluded if they sampled healthy non-clinical populations exclusively, as our focus was on clinicians or patients—individuals within society who have had significant experiences of suffering. While the original study [1] and search strategy herein did not exclude students, in keeping with the iterative nature of scoping reviews [56], the search criteria was refined at the title and abstract screening phase to exclude studies that sampled students exclusively (i.e. nursing students, residents, medical students, etc.), for the sake of feasibility and in recognition that barriers and facilitators to compassion in healthcare primarily occur in ongoing clinical practice [60, 61]. We were interested in studies that had a primary aim to explore compassion towards others within the clinical setting or those that focused on interventions or educational programs aimed at improving compassion in clinical care. As such, studies that focused on other related concepts such as compassion fatigue, compassion satisfaction, empathy, or intervention studies aimed at fostering self-compassion (i.e. mindfulness-based stress reduction or compassion-focused therapies) were excluded. Congruent with the original scoping review [1], we retained a broad interest on categories of studies exploring compassion in healthcare such as perspectives, clinical outcomes, knowledge, skills, or attitudes on the topic [1]. Only primary and secondary studies using qualitative, quantitative, or mixed method designs were included. As such, systematic reviews, books, chapters, letters, commentaries, editorials, dissertations/theses, conference abstracts, and case studies were excluded [1].

### Study selection

At the title and abstract screening (level 1), a calibration exercise of a random sample of articles (*n* = 50) was conducted by two independent reviewers (SM and SS), to test the screening tool to ensure a standardized application of the selection criteria. At level 1, a minimum threshold of 80% agreement (number of agreements/number of agreements + disagreements) [62, 63] was utilized to guide screening of the remaining titles and abstracts. Congruence in the calibration exercise of the 50 articles was 90%, after which one reviewer (SM) proceeded with screening the remaining titles and abstracts [64]. Following the title and abstract screening, two independent reviewers (SM and PJ) conducted a full-text review (level 2) of a random sample of included studies (*n* = 10) to determine whether they would either be included or excluded for data extraction. Congruence in Level 2 screening was initially 70%, with all disagreements being resolved through clarification of the selection criteria and discussion between reviewers until consensus was reached, refining the inclusion/exclusion criteria in an iterative manner, prior to conducting an independent review of the remaining articles that would then proceed to the data extraction phase [65].

### Data items and extraction process

Two reviewers (SM and PJ) independently reviewed each study meeting the criteria for a full-text review to identify eligible studies for data extraction. As an additional measure of rigour and quality assurance, the data extraction form was initially tested between the reviewers for 10 articles, with modification incorporated thereafter [65]. The review team (SM and PJ) met bi-weekly to review the extracted data from each study, resolve any identified discrepancies, and ensure completion and accuracy of the extracted data. A standardized data extraction sheet in excel was used to extract the following variables: study title, author, year published, journal, country of origin, study background and purpose, study setting, design, sample, participant information, data collection methods, analysis methods, results, conclusions, and limitations (both author and reviewer-identified). The manner in which the topic of compassion was conceptualized was also documented for each study.

### Data synthesis

A narrative synthesis of the data was performed given the heterogeneity of studies, in accordance with the original review [1]. Each study was initially grouped by study participants (i.e. HCPs/students or HCPs/patients or patients/students or HCPs/patients/students) and study type (compassion interventions or perspectives of compassion and compassionate behavior). Frequencies for each of these groupings were tabulated. For the narrative synthesis [66], any quantitative data were initially translated to qualitative descriptions. The previously identified categories, themes, and subthemes (Table 1) [1] that emerged from the data in the original scoping review were utilized as a template, allowing us to map the current results onto these pre-existing categories, themes, and subthemes. Any new potential categories, themes, and/or subthemes that emerged were documented and discussed through a consensus process (SM, PJ, SS). Data were analyzed by three members of the research team (SM, PJ and SS), by reviewing the extracted data, resolving any inconsistencies or answering any queries that arose. A decision-making trail was compiled for the placement of the data into their respective categories, themes, and subthemes.

**Table 1:** Categories, Themes, and Subthemes

Categories	Themes	Subthemes
Perspectives on compassion and compassionate behaviour	Nature of compassion	Conceptualizing compassion Temporal Aspects
	Development of compassion	Antecedents of compassion Cultivating compassion
	Interpersonal factors associated with compassion in the clinical setting	Relational factors Clinical communication
	Action and practical compassion	
	Barriers and enablers to compassionate care	Educational barriers Practice setting barriers
	Outcomes of compassionate care	
Compassion Interventions	Clinical interventions	
	Educational interventions	

## Results

### Search flow and study characteristics

**Fig. 2:**
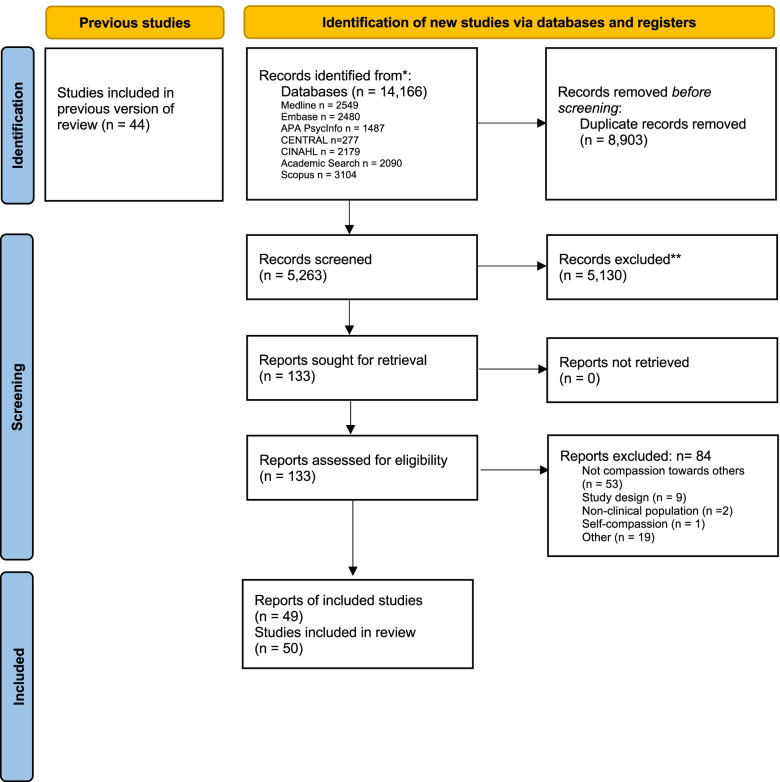
PRISMA 2020 flow diagram for updated systematic reviews which included searches of databases and registers only

Our search strategy resulted in a total of 14,166 records identified from the eight databases (Fig. 2). Removal of duplicates resulted in 5263 records remaining. After title and abstract screening, 133 potentially relevant reports underwent a full text review, after which 84 studies were excluded. A total of 49 articles underwent data extraction and synthesis of results (Table 2) (Fig. 2). One article [67] contained two separate eligible studies and has been reported herein as two separate studies [67, 68]. Thus, for sake of clarity, this narrative synthesis consists of 50 studies. Overall, we found that the studies fell within two overarching categories: perspectives or behaviours of compassion, and compassion interventions (Table 1) [1]. Studies that fell within each of these categories were organized as per their themes and subthemes, according to those that were identified in the original scoping review (Table 3). No new themes or subthemes were identified from the updated search.

**Table 2:** Characteristics of included studies (*n =* 50)*

Characteristic	Number	Percent
**Country**		
United Kingdom (inc. Ireland, Scotland)	19	38
Canada	12	24
United States	6	12
Australia	7	14
Iran	6	12
Norway	3	6
Greece	3	6
Cyprus	3	6
Czech Republic	2	4
Colombia	2	4
Italy	2	4
Hungary	2	4
Israel	2	4
Philippines	2	4
Poland	2	4
Spain	2	4
Turkey	2	4
New Zealand	2	4
**Year**		
2015	3	6
2016	5	10
2017	8	16
2018	11	22
2019	13	26
2020	10	20
**Design**		
Qualitative	35	70
Mixed	5	10
Cross-sectional survey	5	10
Randomized controlled trial (quantitative)	2	4
2 group pre-post	2	4
Delphi or Dillman Approach	1	2
**Setting**		
Education	2	4
Outpatient	3	6
Inpatient and outpatient	2	4
Healthcare organization	1	2
Hospital	7	14
Acute care	1	2
Mother and Baby Unit	1	2
Medical-surgical	2	4
Radiology	1	2
Palliative Care, Hospice	7	14
Long term care	1	2
Critical care and palliative care	1	2
Intensive care	2	4
Rehabilitation Units	1	2
Various (i.e. hospital, community, homecare / acute, home, hospice, residential / internal, surgical, emergency, burn, CCU and ICU / University & Hospital / ICU and pall care / Mental health & acute / primary care)	8	14
Not specified	10	20
**Sample**		
Patients (exclusively)	12	24
Healthcare providers (exclusively)	27	54
Various	16	42.1
Nurses	11	28.9
Healthcare providers and Other (i.e. Physicians and/or Patients and/or Family Caregivers and/or students and/or residents and/or public members)	11	22

*One article by Henshall et al. (2017) contained two eligible studies and have been reported herein separately [67, 68]

**Table 3:** Summary of studies that explored either perspectives of compassion or compassion interventions in healthcare

Study	Country	Participants	Study Design	Setting	Category (P or CI)*	Perspectives										Compassion Interventions	
						Nature of Compassion		Development of Compassion		Interpersonal compassion-ate care		Outcomes of Compassionate care	Action and Practical Compassion	Challenges and Enablers		Clinical	Educational
						**Conceptualizing**	**Temporal**	**Antecedents**	**Cultivating**	**Relational Factors**	**Clinical Communication**			**Educational**	**Practice Setting**		
**Healthcare Providers**																	
Saab et al. [69]	UK (Ireland)	Healthcare providers (*n =* 79) *(Various)*	Quantitative survey design	Hospital - Nurse and midwifery unit	CI												X
MacArthur et al. [70]	UK	Stakeholders: Charge nurses and nurse managers (*n =* 14); Practitioners: Senior nurses within the LCC programme (*n =* 7); Policy makers: Senior individuals in the NHS organization and higher education institution (*n =* 5) *(Various)*	Qualitative	Beacon wards and developmental sites: acute care, longterm care, mental health, palliative care.	CI												X
Smith et al. [71]	UK (Scotland)	Healthcare providers and managers (*N =* 132) *(Various)*	Mixed methods	8 NHS Boards	CI												X
Roze des Ordons et al. [72]	Canada	Healthcare providers (*n =* 5) *(Various)*	Qualitative	Critical and Palliative care	P				X	X	X		X	X	X		
Singh et al. [73]	Canada	Clinicians (*n =* 57) *(Various)*	Qualitative	Palliative care	P			X		X			X		X		
Valizadeh et al. [74]	Iran	Nurses (*n =* 15)	Qualitative	Hospital	P									X	X		
Ling et al. [75]	Australia	Healthcare providers (*n =* 75) *(Various)*	2 group pre-post	Epworth HealthCare organization	P				X								
Jones et al. [76]	Australia	Nurses (*n =* 171)	Qualitative	Intensive care	P			X		X					X		
Papadopoulos et al. [77]	Australia, Colombia, Cyprus, Czech Republic, Greece, Hungary, Isreal, Italy, Norway, Philippines, Poland, Spain, Turkey, UK, USA	Nurses (*n =* 1323)	Mixed – Survey design		P	X		X		X			X		X		
Murray et al. [78]	Australia	Nurses and midwives (*n =* 50) *(Various)*	Qualitative	8 healthcare facilities	P					X					X		
Brennan et al. [79]	UK	Healthcare providers (*n =* 12) *(Various)*	Mixed	Outpatient daycentre services	P	X	X	X		X	X				X		
Sinclair et al. [45]	Canada	Healthcare providers (*n =* 57) *(Various)*	Qualitative	Hospital palliative care unit; hospital and rural palliative care consult service; hospice; outpatient oncology palliative care	P	X		X		X	X		X				
Zamanzadeh et al. [80]	Iran	Nurses (*n =* 16)	Qualitative	Hospital	P			X	X								
Bessen et al. [81]	USA	Healthcare Providers (*n =* 13) *(Various)*	Qualitative	Palliative care; Hematology/Oncology	P	X	X						X		X		
Papadopoulos et al. [82]	Australia, Colombia, Cyprus, Czech Republic, Greece, Hungary, Isreal, Italy, Norway, Philippines, Poland, Spain, Turkey, UK, USA	Nurses (*n =* 1323)	Cross-sectional survey (quantitative)	NR	P	X			X								
Roze des Ordons et al. [83]	Canada	Healthcare providers (*n =* 5) *(Various)*	Qualitative	ICU and Palliative care	P	X	X		X	X			X				
Tierney et al. [84]	UK	Healthcare providers (*n =* 36) *(Various)*	Qualitative	Diabetes-specific NHS Trusts	P	X		X		X	X		X		X		
Ledoux et al. [85]	Canada	Nurses (*n =* 191)	Quantitative (Dillman Approach)	Hospital (Inpatient or Outpatient)	P			X		X					X		
Efstathiou et al. [86]	UK	Nurses (*n =* 12)	Qualitative	ICU	P	X					X		X				
Aagard et al. [87]	USA	Nurses (*n =* 50)	Qualitative Survey design	NR	P	X	X			X	X		X				
Henshall et al. [67]	UK	Healthcare providers (*n =* 276) *(Various)*	Cross-sectional survey (Quantitative)	Mental health; Acute care	P			X							X		
Henshall et al. [68]	UK	Healthcare providers (*n =* 276) *(Various)*	Cross-sectional survey (Quantitative)	Mental health; Acute care	P										X		
Papadopoulos et al. [88]	Greece and Cyprus	Nurses (*n =* 143)	Mixed – Survey design	NR	P	X		X	X	X	X		X		X		
Skorpen Tarberg et al. [89]	Norway	Nurses (*n =* 21)	Qualitative	Primary care and Nursing homes	P	X	X			X	X		X		X		
Burridge et al. [90]	Australia	Nurses (*n =* 20)	Qualitative	Rehabilitation Units (Spinal Injury and Brain Injury)	P	X	X	X		X					X		
Ferraz et al. [91]	Australia	Healthcare providers (*n =* 18) *(Various)*	Qualitative	Hospital and community based – pediatric and adult palliative care settings	P	X				X	X				X		
**Healthcare Providers, students**																	
Christiansen et al. [92]	UK	Healthcare providers (*n =* 155), Healthcare students (*n =* 197) *(Various)*	Qualitative	University-registered	P	X				X					X		
Reynolds et al. [93]	New Zealand	Healthcare providers (*n =* 108), medical students (*n =* 219) *(Various)*	Qualitative	Experimental lab conditions in the medical context	P				X	X							
Uygur et al. [94]	Canada	Family physicians (*n =* 16), residents (*n =* 6)	Qualitative	Inpatient and outpatient settings	P		X	X	X	X	X	X			X		
**Healthcare Providers, students, public**																	
Kneafsey et al. [95]	UK	Social care staff and students, members of the public (*n =* 45)	Qualitative	Hospital; University	P	X	X	X	X	X	X		X		X		
**Healthcare Providers, patients, family caregivers**																	
Sims et al. [96]	UK	Healthcare providers (*n =* 50), Patients (*n =* 34), Family carers (*n =* 28)	Qualitative	Acute care: orthopaedic, surgery, cardiology and respiratory medicine	P					X	X				X		
Gould et al. [97]	UK	Healthcare providers (*n =* 166), Patients (*n =* 273)	2 group pre-post (quantitative)	Medical and surgical	CI											X	
Taylor et al. [98]	UK	MRTs (*n =* 27) SMRTs (*n =* 24) PaCs (*n =* 16), Patients (*n =* 11), caregivers (*n =* 5)	Qualitative	3 NHS Trusts	P	X		X		X	X				X		
Tehranineshat et al. [99]	Iran	Nurses (*n =* 20), Patients (*n* = 8), Family caregivers (*n =* 6)	Qualitative	Internal, surgical, emergency, burn, CCU, and ICU	P					X	X		X				
Babaei et al. [100]	Iran	Nurses (*n =* 40), patients (*n =* 16), and family members (*n =* 8)	Qualitative	Medical-surgical	P				X						X		
Smith-Macdonald et al. [101]	Canada	Healthcare providers (*n =* 72), managers (*n =* 9), longterm care Residents (*n =* 20), family members (*n =* 16)	Qualitative	Longterm care	P	X		X	X	X	X		X		X		
Babaei et al. [102]	Iran	Nurses (*n =* 20), patents (*n =* 8), family members (*n =* 4)	Qualitative	Hospital: Adult general wards (6 internal and 4 surgery)	P	X		X		X	X				X		
**Patients**																	
Fernando et al. [103]	New Zealand	Advanced palliative care patients (*n =* 20)	Qualitative	Palliative care	P	X				X	X		X				
Tanco et al. [104]	USA	Cancer patients (*n =* 100)	Randomized Clinical Trial (quantitative)	Hospital outpatient clinic	P						X						
Tanco, et al. [105]	USA	Advanced cancer patients (*n =* 128)	Randomized Clinical Trial (quantitative)	Outpatient supportive cancer centre	P						X						
Straughair et al. [106]	UK	Patients with personal experience with nursing care (*n =* 11)	Qualitative	Hospital, community, homecare	P	X		X	X	X					X		
Sinclair et al. [50]	Canada	Adult cancer inpatients (*n =* 53)	Qualitative	Hospital palliative care unit and palliative care consult service	P			X	X	X	X		X	X			
Dalvandi et al. [107]	Iran	Patients (*n =* 300)	Cross-sectional survey (quantitative)	Hospital - Internal and surgical wards	P	X				X			X	X			
Wittkowski et al. [108]	UK	Patients (i.e. mothers admitted to the Mother and Baby Unit) (*n =* 17)	Mixed Methods	Mother and Baby Unit	P					X	X		X				
Sinclair et al. [6]	Canada	Adult cancer inpatients (*n =* 53)	Qualitative	Hospital - Palliative care unit and hospital palliative care consult service	P	X	X	X		X	X	X	X				
Menage et al. [109]	UK	Women patients who self-identified as recipients of compassionate midwifery care during their pregnancy (*n =* 17)	Qualitative	NHS Trusts	P	X		X		X	X		X		X		
Sinclair et al. [44]	Canada	Advanced cancer inpatients (*n =* 53)	Qualitative	Hospital – Palliative care	P	X		X		X		X	X				
Sinclair et al. [110]	Canada	Patients with life limiting illnesses (*n =* 20)	Qualitative	Acute care; Longterm care; Hospice; Home care	P		X	X		X	X		X				
Singh et al. [111]	Canada	Patients (*n =* 19)	Qualitative	Acute care	P	X		X		X	X	X	X		X		
**Patients, students**																	
Bleiker et al. [112]	UK	Patients (*n =* 34) Medical imaging professional undergrad students (*n =* 6)	Qualitative	Radiology	P	X		X	X	X	X		X		X		

*P Perspectives, *CI* Compassion Interventions

Studies were predominantly qualitative in nature, with some quantitative and mixed-methods study designs. Two studies were randomized controlled trials (quantitative design). Most of the studies were conducted in the United Kingdom (Table 2), followed by Canada and the United States. Two studies collected data from 15 different countries (Table 2), with two other secondary studies utilizing this larger dataset to report exclusively on the results from Greece and Cyprus, and the USA exclusively. Twelve studies sampled patients, and the remainder focused on HCP participants or a mix of HCP, students, patients, and/or family caregivers (Tables 2 and 3).

### Category: perspectives on compassion and compassionate behaviours in healthcare

Forty-six studies explored perspectives on the nature of compassion or compassionate behaviours (Table 3). Similar to the previous scoping review [1], perspectives on compassion were presented from either patients or HCPs, or a combination of participants (i.e. HCPs and/or patients along with students, family caregivers or even the public) (Table 3). The majority of these studies presenting perspectives on compassion were qualitative in design (*n* = 35), followed by mixed methods (*n* = 5) and cross-sectional survey studies (*n =* 5). Two were quantitative randomized clinical trials. Twenty-three studies on perspectives of compassion sampled HCP participants exclusively (Table 3).

### Theme: the nature of compassion in healthcare

Twenty-seven studies reported participants’ perspectives on the nature of compassion, which included the conceptualization of compassion and/or its temporal aspects (Table 3).

### Subtheme: conceptualizing compassion in healthcare

Compassion was conceptualized through patient perspectives (*n* = 12 studies), in which participants were asked about what compassion meant to them in reflecting on their personal experiences with their HCPs [6, 45, 98, 101–103, 106, 107, 109–112]. Several features that patients recognized as signifying compassion included: kindness, authenticity, attentiveness, forming a relational connection, displaying presence and warmth, acceptance, understanding, listening, helping, communicating effectively, being involved, and being gentle and caring [6, 44, 98, 101–103, 106, 107, 109–112]. Sinclair et al. (2016; 2018) conceptualized and validated an empirical model of compassion from both the patient and HCP perspective, further defining compassion as “a virtuous response that seeks to address the suffering and needs of a person through relational understanding and action” [6, 45]. Menage et al. (2020) also conceptualized compassion in midwifery care through a model, highlighting its key components as “being with me”, “relationship with me”, and “empowering me” [109]. International, online survey studies conducted with a total of 1323 nurses, nurse educators, and nurse managers representing 15 different countries (Table 3) used a pre-imposed dictionary definition of compassion “a deep awareness of the suffering of others and a wish to alleviate it” [77, 82, 87, 88]. Interestingly, while this survey defined compassion a priori [77, 82, 87, 88], some participants provided their own definitions, with some participants from Spain identifying this definition as problematic, noting that the term compassion itself was problematic in being associated with religious beliefs, and as such, diminishing the evidence-based approach of nursing care within the Spanish context [77]. One study aimed to investigate compassion in a specific cultural context focusing particularly on South Asian patients [111]. While South Asians perceived compassion in a similar vein to other patient groups (i.e. compassion being composed of HCP embedded qualities, relational connection, and an action-orientated nature) [6, 110] and as a universal concept that extends across humanity regardless of cultural differences, they also highlighted the importance of compassionate HCPs possessing cultural sensitivity, and accepting cultural beliefs and practices in a non-judgemental manner [111]. In another study, patient participants felt that compassion was demonstrated through HCPs’ ability to demonstrate intuition, provide evidence-based care, and be proficient in managing time in their clinical practice [99]. Similarly, Dalvandi et al. (2019) reported that patients perceived compassion to be associated with a capable HCP [107]. In fact, the authors reported that HCPs’ caring attributes or ability to meaningfully connect with his/her patients was seen as less desirable compared to their overall clinical competence [107].

Eighteen studies highlighted HCP conceptualizations of compassion and compassionate behaviours [45, 77, 79, 81–84, 86–92, 95, 98, 101, 102]. Similar to patients, HCPs recognized compassion as involving an inner desire to want to relieve one’s suffering [45, 79, 87] and as a response based on sensitivity to patients’ preferences [90]. Sinclair et al. (2018) generated a HCP model of compassion, in which HCPs defined compassion as a “virtuous and intentional response to know a person, to discern their needs and ameliorate their suffering through relational understanding and action” (p. 5) [45]. Additionally, in a study by Tierney et al. (2017), HCPs defined compassion in the workplace as “professional compassion,” which encompassed traits such as being a good communicator, being cognizant of patients’ needs, and through providing small acts of kindness [84]. This study also described the concept of professional compassion as involving some degree of “tough love” or through providing “scare tactics” (i.e. emphasizing to patients their medical conditions that may result in their mortality), which was driven from practitioners’ desire to help prevent future medical complications in patients – an approach to care that was further emphasized in relation to compassion sometimes requiring a more conscious effort as opposed to it occurring spontaneously [84]. In studies involving physicians from palliative care and medical oncology contexts [81, 89], while compassion was thought to consist of both intangible and tangible skills (i.e. being present, holding a patient’s hand, and supportive touch) to address patients’ emotional needs [81], having standardized end-of-life conversations with patients and their family caregivers was integral to ensuring that their needs were adequately addressed and to educating them about their disease trajectory [81, 89].

### Subtheme: temporal aspects of compassion in healthcare

Patient and HCP participants alike perceived time as one of the components related to the nature of compassion, describing compassion as being fluid and dynamic in nature, something that can be developed over time, while also being influenced by the availability of time (i.e. taking time to listen to patients) [6, 81, 83, 87, 94, 95, 110]. Although compassion has been reported as developing over time, it has also been recognized as something that can be attained by HCPs in instances where there is a limited amount of time, through thoughtfully connecting with patients in the moment, acknowledging the difficulties that they’re facing, using humor, physical gestures conveying comfort, and relating to patients’ social concerns [81, 94]. Compassion was recognized as adaptable to the situation and clinical setting [83, 90] and something that patients may better appreciate and become more aware of overtime during their care journey [90]. Interestingly, nurse participants in the rehabilitation setting thought that providing compassion should not be obligatory given its situational nature and should instead be delivered with discretion or tempered [90]. As such, the provision of compassion depended on the individual nurse’s own personal values balanced with their duty of care [90]. Compassion was also perceived as requiring HCPs to “slow down” [83, 89], particularly in the palliative care context where creating a space for dying was characterized by ‘slowness’ [89].

### Theme: the development of compassion in healthcare

Thirty studies explored the development of compassion, which included both its innate nature and external factors that could equip clinicians with the necessary skills to further enact compassion in their clinical care.

### Subtheme: antecedents of compassion

Patients and HCPs both recognized the intrinsic qualities or virtues of individual HCPs to be integral to providing compassion, some of which included virtues of love, kindness, genuineness, consideration, understanding, and wisdom [6, 44, 45, 50, 68, 73, 77, 79, 80, 82, 84, 85, 88, 90, 94, 95, 98, 101, 102, 106, 109–112] (Table 3). While various participants in a study by Kneafsey et al. (2015) described compassion as an innate emotion and a part of one’s personality at birth [94, 95], other studies recognized that past experiences also shape one’s ability to be compassionate [76, 80, 85]. For example, nurses described their own psychological empowerment to contribute to their ability to provide more compassion, as driven by their length of experience working within the field of healthcare [85]. Patients and clinicians also perceived compassion to be motivated by their own personal experiences of suffering, having had to provide care to ill family members, or to be developed through family upbringing, role modelling, self-reflection and life experiences [6, 76, 80, 110]. Religion, spirituality or culture, and an appreciation for a recognition of the shared humanity between oneself, patients, family, and colleagues were also perceived as external factors that could facilitate or motivate one to provide compassion [80, 90, 110, 111]. Furthermore, various HCP participants in a study by Taylor et al. (2020) indicated that their own cultural values must denote they are person-centred, caring, and open, and hold intent to be compassionate [98]. Additionally, a HCP’s attitude was found to be a forerunner of compassion, influencing their behaviour and practices towards their patients [98, 102]. In a few studies, HCP participants felt that compassion should be a prerequisite to pursuing a career in healthcare [79, 80, 84, 94]. In one study, HCP participants felt that a personal interest in compassion must be vested in, dismissing the notion of any external motivations or conditions compelling one to being compassionate (i.e. from the healthcare organization itself) [80]. In other studies, participants described compassion as a predisposition or a driver through which they chose to pursue a career in healthcare and a core value that draws many physicians and nurses into healthcare professions [84, 94].

### Subtheme: cultivating compassion

Several studies reported that compassion can be taught and that HCPs can be equipped with tangible knowledge and skills for improving compassion in their professional practice [50, 82, 88, 94, 95, 101, 106]. Both HCP and patient participants emphasized clinical role-modeling, by compassionate HCPs, as being a salient means for improving compassion [50, 72, 80, 83, 100, 106]. While role models in the form of teachers or peers were seen as imperative for motivating physicians and nurses (especially newly graduated clinicians) to be compassionate [80, 94], it was also suggested that uncompassionate behaviours (i.e. answering to patients indifferently, ambivalence, and disregard) were equally transferrable to HCP colleagues [100]. A supportive environment that is conducive to learning was another factor that patients thought to be advantageous in compassion training [50]. Patients receiving palliative care believed that HCPs might increase their capacity for compassion by adopting a more reflective practice – through contemplating their own beliefs and reflecting on what their patients may be experiencing [50] – an experiential approach that was further supported by patient and HCP participants in a study by Smith-MacDonald et al. (2019) [101].

Reynolds et al. (2019) explored the effectiveness of “compassion-inducing” images to combat clinical scenarios that were thought to challenge medical students’ and health professionals’ (physicians, nurses, other) ability to be compassionate in dealing with patients presenting with “disgusting symptoms” and/or those who were thought to be responsible for their own health problems [93]. The authors reported that while patients presenting with “disgusting symptoms” (i.e. more challenging, more likely to wear a mask) influenced medical students more than the qualified HCPs, the use of compassion-inducing images mitigated group differences [93]. Similarly, Ling et al. (2020) tested the impact of “common humanity scenarios” on one’s ability to provide compassion and found that study participants (nursing, medicine, social work, occupational therapists, pastoral care practitioners, etc.), reported enhanced levels of compassion after being exposed to scenarios reflecting common humanity, further identifying common humanity as a prerequisite for providing compassion [75].

### Theme: interpersonal factors associated with compassion in the clinical setting

Both relational and clinical communication were the predominant interpersonal dimensions associated with compassion, identified in studies involving both HCP and patient participants.

### Subtheme: relational factors

The ability of HCPs to interact with patients, to deeply connect and share in their experience through an outward expression of their innate virtues, along with creating a relational space to do so, was seen as paramount to the provision of compassion in healthcare. This space was commonly described by patients as extending beyond a clinical relationship to one in which HCPs would actively engage in the patients’ suffering through awareness and engaged caregiving [6, 44, 45, 73, 91, 101, 110, 111]. This entailed not only being physically present with the patient and addressing their medical needs, but seeking to understand their unique needs (e.g. emotional) and appreciate the patient as a person [6, 44, 45, 73, 79, 89, 99, 101, 110, 111]. An inability to understand the emotional state of the patient or leaving patients feeling worried or vulnerable was felt to be associated with uncompassionate HCPs [91, 94, 99, 102]. Skills such as being able to express affection, kindness, tenderness, being able to actively listen [77, 78, 88, 89, 94], showing understanding, and being supportive were perceived to be more effective expressions of compassion than routine, task-oriented care [99]. The ability to relationally understand patients was further highlighted as a distinct feature from sympathy, in which a shallow and superficial emotional response from HCPs can leave patients feeling demoralized, depressed, and feeling pity for themselves [44]. Getting to know the patient and going through a process of knowing through recurrent interactions and building rapport was important to both patients and HCPs alike [84, 87, 98, 112]. Study participants indicated that encounters which lack connection renders HCPs as ingenuine and as having a lack of compassionate intent [98]. HCPs highlighted relational challenges such as receptivity, proximity, fragmentation, and lack of shared understanding between and within the healthcare team(s) and patients as potential hinderance to compassion [72]. On the contrary, being able to build rapport and connect with or relate to patients and their family was perceived as essential [76]. As such, relational aspects of compassion were found to be multidirectional, expressed between two or more people including patients, families, and HCPs [83]. Inside the workplace, supportive inter- and intra-disciplinary relationships helped to enhance the unity of the care team and thus aid in the development of more concise care plans which led to more consistency in patient care [76, 102]. A few studies noted some subtle differences in the expressed relational needs of females versus males, which may impact compassion. For example, female participants emphasized their emotional needs requiring more attention in comparison to men [107] and thus, reflecting the need for female nurse HCPs to be able to better relate to them and subsequently personalize their care more effectively [108, 109].

### Subtheme: clinical communication

Both patient and HCP participants identified clinical communication to be a prominent component of compassion in healthcare. An integral domain of the Patient Compassion Model [6, 110] was relational communication, referring to the verbal and non-verbal displays of compassion within the clinical context that seeks to establish a deeper understanding of a person as an individual – an aspect of compassion that was also identified in other studies [6, 45, 101, 110]. Facets of relational communication included HCPs demeanor, affect (emotional resonance), engagement and behaviour [6, 45, 101, 110]. It was also stated in one study that through relational communication, actively listening, involved listening to the subtext of what is not said (i.e., being attuned to the non-verbal cues, using silence, and paying attention to tone of voice), can help uncover patients’ concealed suffering [45]. Taylor et al. (2020) further differentiate that indicative communication is different from traditional communication, such that body language must be accompanied with tone of voice to demonstrate a compassionate intent [98].

Other studies have also highlighted notable components of verbal and non-verbal communication in which HCPs can convey compassion to their patients, such as tone of voice, personalization, attentiveness, actively listening, body language (i.e. smiling, eye contact) and even touch [79, 87–89, 91, 94, 95, 99, 109]. While communicating relevant information in a timely manner was seen as paramount to providing compassion for patients undergoing withdrawal of life-sustaining treatment [86], it was also found that HCPs’ display of emotion could help further humanize the interaction with patients, as long as it was exhibited in ways that were not too burdensome to the patient’s family [86]. Patients’ perception of compassion may also depend largely on the content that is being delivered [72, 104, 105]. For example, using the Physician Compassion Questionnaire to rate clinicians on five dimensions of warm-cold; pleasant-unpleasant; compassionate-distant; sensitive-insensitive; and caring-uncaring [104], advanced cancer patients in a randomized clinical trial considered physicians who provided a more optimistic prognosis to be significantly more compassionate compared to physicians who delivered less optimistic prognostic information [104, 105]. Similarly in another study, HCP participants felt that despite a HCP’s intention, more emotionally challenging conversations involving goals of care or prognostication may be perceived by patients as being less compassionate [72]. Various studies suggest that communicating information through plain and simple language for patients to understand, delivered in a sensitive manner, was crucial in demonstrating compassion [6, 95, 103, 110]. Also, women participants who received midwifery care during their pregnancy perceived midwives as more compassionate when they “communicate as an equal” – i.e. without any implied professional superiority [109].

In two cross sectional survey studies, in addition to other modes of non-verbal communication such as listening and connecting with patients, nurse participants particularly highlighted “touching” (i.e. holding a patient’s hand, giving a back rub, placing a hand on the patient, healing touch, or human touch) to be paramount to relieving one’s suffering and offering a sense of healing and comfort [87, 88]. Along with having HCPs identify with patients by trying to understand their situation, patients and HCPs in various studies emphasized supportive touch to be an important aspect of HCPs connecting with the patients [45, 50, 83, 101, 103].

### Theme: action and practical compassion in healthcare

Patients and HCPs stipulated action-based components of compassion as quintessential, particularly those directed at proactively alleviating patient suffering and addressing their needs through tangible means [6, 44, 45, 50, 73, 81, 95, 101, 110, 111], whilst considering sensitivity to the patients’ condition [6, 45, 103]. Participants referred to the importance of “small acts of kindness” across a few studies [6, 44, 45, 72, 83, 84, 103, 110], such as providing comfort [86, 87, 103] and performing actions that were supererogatory in nature or going above and beyond without expectation of receiving anything in return, as key features of actions associated with compassion [6, 77, 81, 87, 110]. For nurses in palliative care, action was evident in being proactive in planning the palliative pathway with the patients and families before the patient had reached their terminal phase [89]. In some cases, the technical and physical aspects of compassion compared to other humanistic approaches were more desirable to patients, particularly those in hospital surgery wards, where the alleviation of pain is more critical to their overall health quality [107], or when looking for compassion in the task-based features of having to undergo diagnostic tests, such as radiography [112]. In contrast, clinicians in a study conducted by Roze des Ordons et al. (2020) felt that an overly biomedical approach could contribute to the over medicalization of an illness which in turn can lead to more patient suffering and even missed opportunities for integrating patients’ goals and values into their care plans, which is paramount in improving quality of life [72]. Similarly, nurses in one study felt that to be compassionate, there is a strong need for competency in relieving pain through both pharmacological and non-pharmacological means [87], including but not limited to, providing emotional comfort. Finally, in addition to utilizing a proactive action-based approach to care, patients receiving midwifery care felt that a midwife’s ability to teach and coach mothers by providing them with necessary information about their condition was considered an act of practical compassion [109].

### Theme: challenges and enablers of compassion in healthcare

More than half of the studies (*n* = 30; 60%) identified various educational and/or practice-setting challenges and enablers to compassion (Table 3), with 11 studies specifically identifying the exploration of barriers and facilitators to compassion in hospital, critical care, palliative care, intensive care, mental health, acute care, long-term care, and medical-surgical contexts as a primary study aim [67, 68, 72–74, 76, 84, 92, 100–102].

### Subtheme: educational challenges and enablers

Four studies identified educational challenges and barriers to providing compassion [50, 74, 83, 107]. HCP participants identified feeling particularly challenged in providing compassion within clinical practice, when there was a perceived incongruence between their theoretical knowledge of compassion and their ability to apply it [72, 74]—a phenomena conceptualized as compassion distress [44]. Similarly, while clinicians acknowledged that they could learn compassion vicariously through their colleague role models (i.e. enablers to compassion), not having those role models routinely available impeded HCPs’ ability to grow in their capacity for compassion [72]. Patients, on the contrary, felt that a supportive teaching environment was necessary to allow HCPs to safely reflect on their innate qualities, such as their virtuous, past life experiences, and vocational motivators to further nourish their abilities to provide compassion [50]. Patients in this study also felt that experiential methods of learning compassion would likely be more beneficial to HCPs over traditional didactic approaches [50]. Interestingly, in contrast to HCP education in providing compassion, a survey conducted with 300 hospitalized patients that aimed to determine the importance and extent of providing compassion in nursing care revealed that patients level of education influenced how compassion was experienced – i.e. patients with an academic-level of education were more aware of system issues, had better communication skills and a higher expectation to participate in the treatment process, and as such perceived HCPs as being less compassionate than those who had lower than a diploma level of education [107].

### Subtheme: practice setting challenges and enablers

Numerous studies (*n* = 29) identified specific challenges and enablers impacting the provision of compassion within the healthcare setting, with challenges being identified disproportionally in comparison to enablers. The most commonly identified challenges were time constraints [72–74, 77, 79, 81, 84, 88, 91, 92, 95, 96, 101, 106, 112], organizational culture (i.e. excessive workload and inadequate staffing) [72, 73, 77, 79, 84, 88, 90, 92, 94, 96, 100, 106, 109, 112], lack of resources [79, 100], and the clinical environment/culture itself [72, 74, 79, 89, 90, 92, 94, 95, 98, 100, 101, 106, 112]. Some studies commented on how advancing technology in the clinical setting can serve as a barrier to HCPs’ ability to provide compassion [72, 106]. For example, HCPs in critical and palliative care settings perceived technology as distracting them from attending to their patients’ emotional needs, requiring them instead to focus more on physical aspects of care [72]. HCP and patient participants in other studies felt that the need for HCPs to juggle daily administrative or organizational requirements contributed to a myopic focus of care that centred on tasks or checking off “ticky boxes” rather than on providing high-quality, compassion [77, 109]. One study identified organizational threats (i.e. daily organizational demands and workplace stresses) as inhibitors to compassion [67], where increased perceived organizational threats led to a decreased ability for HCPs to provide compassion to patients [67]. Sims et al. (2020) further examined “intentional rounding”, a structured process that involves nurses performing periodic checks of their patients’ fundamental care needs using a standardised protocol and documentation, and its contribution to the delivery of compassionate nursing care. Ironically, this care strategy was actually perceived by participants to be more of a barrier to providing compassion, as nurses were left to prioritize their documentation over direct patient care [96].

Another practice setting barrier to providing compassion was the lack of managerial engagement or support [74, 101], which can contribute to fragmented teams, lack of unity [76, 101], resulting in less compassion to patients in settings were collaboration between nurses was lacking [89]. Additionally, nurses in hospital settings felt muted in their ability to provide compassion when their managers failed to support them in its delivery [74]. HCPs also felt that after the death of a patient, their grief and mourning affected their ability to provide compassion to their patients, highlighting the need for managerial support and compassion towards themselves from their colleagues or managers as they worked through their own mourning [98]. In the acute care context, care was thought to be susceptible to fragmentation given the various division of HCP roles, multiple team members, shiftwork, and sequential transitions, all leading to varying intensity and duration of patient interactions [72]. On the other hand, support networks amongst HCPs were also seen as enablers to compassion within various practice settings [68, 76, 78, 79, 91, 98]. For example, the need for HCPs to engage in a team dynamic to support the delivery of compassion to patients was highlighted by Murray et al. (2020), specifically with respect to maintaining good communication, encouraging and listening to one another, being present, and open-minded [78]. Findings from Brennan et al. (2019) concur with this notion of the importance of HCPs fostering strong connections with their colleagues to enhance the delivery of compassion within their organizational settings [79].

In general, literature on clinical challenges and enablers from the patient perspective appeared to be lacking. Ironically, the majority of studies focused on HCPs perspectives on patient factors (personality, behaviours, communication issues, etc.) effecting HCPs ability to provide compassion in clinical care, with little discussion of HCP factors (personality, behaviours, communication issues, etc.). Studies that did include patients’ perspectives, identified language barriers as a significant challenge to experiencing compassion from their HCPs [72, 100, 102, 111], reportedly undermining HCPs’ motivation or aptitude for providing compassion in the process [100, 111]. However, a study by Singh et al. (2020), acknowledged that language barriers could be overcome by having interpreters readily available and by being cognizant of patients, particularly female patients, preferences related to the sex and gender of their HCPs [111]. Interestingly, in a qualitative study of nurses, family members and patients, sex was also a predictor of compassion, with women being perceived as being more innately compassionate than men [100].

### Theme: outcomes of compassionate care

Three studies identified the impact of compassion on patient health outcomes exclusively from the patient perspective [6, 44, 111], and one from the perspectives of both family physicians and inpatient/outpatient residents [94]. Patients felt that compassion alleviated their suffering, enhanced overall well-being, and positively augmented the quality of care they received from their HCPs by allaying distress and enhancing their relationship with their HCPs [6, 44, 111]. On the contrary, those patients who recoined healthcare interactions lacking in compassion reported negative outcomes such as frustration, being overwhelmed, and a lack of dignity and hope [6]. In a separate study, patients felt that compassionate physicians achieve a better understanding of their patients’ issues and concerns, facilitating more open communication, which in turn helps to strengthen the level of trust in the patient-physician relationship [94]. Compassion was also felt to have assisted physicians in constructing more supportive and caring treatment plans for their patients, which ultimately facilitated patient compliance [94]. Lastly, a compassionate approach was perceived to help enable physicians to better cope with more challenging patient scenarios, such as those patients presenting with more psychosocial or emotional distress [94].

### Category: compassion interventions

Four studies focused on compassion interventions for HCPs (i.e. clinicians, policy makers, and managers) and patients (Table 4). These interventions studies traversed the themes of clinical and educational interventions (Table 3).

**Table 4:** Studies that implemented compassion interventions

Study	Country	Setting	Design (evaluation)	Intervention	Participants	Outcomes (improved)
Saab et al. [69]	UK (Ireland)	Hospital – Nurse and midwifery unit	1 group (quantitative – survey)	Leaders for Compassionate Care Programme (LCCP): To empower leaders while supporting their teams in delivering high-quality and compassionate patient-centered care.	Clinical nurse/midwife manager (*n =* 73); Clinical nurse/midwife specialist (*n =* 3); Assistant director nursing/midwife (*n =* 2); Advanced nurse/midwife practitioner (*n =* 1)	The Leaders for Compassionate Care Outcomes Evaluation Questionnaire (LCCOEQ) that measured outcomes related to 4 domains of leadership practice: (1) Understanding of context (yes) (2) Introduction to skills in quality improvement and management of change (yes) (3) Personal development (yes) (4) Relational development (yes)
MacArthur et al. [70]	UK	Beacon wards and developmental sites: acute care, long-term care, mental health, palliative care.	1 group (qualitative)	Leaders for Compassionate Care Programme (LCCP): To embed and sustain a culture of compassionate care within the reality of modern health care environments	Stakeholders: Charge nurses and nurse managers (*n =* 14); Practitioners: Senior nurses within the LCC programme (*n =* 7); Policy makers: Senior individuals in the NHS organization and higher education institution (*n =* 5)	Relationships – changes between groups and individuals over time as a result of the exploration of the meaning of compassionate care and the introduction of methods for giving and receiving feedback (yes) Care delivery – new approaches, attitudes and behaviours influenced by practice development techniques that placed emphasis on values and expression of emotions (yes) Developments in practice – specific action projects that had been initiated by staff as a result of the action research elements of the LCC Programme (yes)
Smith et al. [71]	UK (Scotland)	Not reported	1 group longitudinal (Mixed methods; 2 phases)	Valuing Feedback (VF) Programme: To develop learning materials focused on gathering feedback to support the development of compassionate care practice.	Healthcare providers and managers (*N =* 132).	Phase 1: evaluation questions during the three sessions conducted in each participating NHS Board Phase 2: online survey and a semi-structured telephone interview to determine the impact of the programme on participants’ practice over time
Gould et al. [97]	UK	Medical and Surgical Wards	2 group (intervention or control) pre-post (quantitative)	Creating Learning Environments for Compassionate Care (CLECC): To embed ward-based manager and team practices including dialogue, reflective learning and mutual support to enhance team capacity to provide compassionate care.	Patients (*n =* 273); Healthcare assistants (*n =* 74), staff nurses (*n =* 74), and sisters/charge nurses (*n =* 18)	Quality of Interactions Schedule (QuIS) on staff-patient interactions (yes) Patient-reported Evaluation of Emotional Care during Hospitalisation (PEECH) tool on emotional care (yes) Jefferson Scale of Empathy (JSE) (Physician / HP version) (no)

### Theme: clinical interventions

Gould et al. (2017) conducted a quantitative (baseline and 4 months post) intervention with clinicians (ward managers, healthcare assistants, staff nurses and charge nurses) and patients [97]. This study sought to evaluate the “Creating Learning Environments for Compassionate Care (CLECC)” program that aimed to enhance clinicians’ capacity for providing compassion by embedding ward-based manager and team practices including dialogue, reflective learning, and mutual support [97]. As indicated by patient-reported evaluations of emotional care using the Patient Evaluation of Emotional Care during Hospitalisation (PEECH) tool, higher scores post-intervention indicated better patient-reported experiences. However, staff self-reported empathy, using the Jefferson Empathy Scale, did not show any significant difference in scores between baseline and follow-up. Overall, the CLECC program was favorable towards reducing negative staff-patient interactions and was anecdotally felt to offer potential benefit in reducing patients’ experiences of lack of emotional connection with the healthcare team [97].

### Theme: educational interventions

Three studies were thematized as educational interventions, each of which were components of the Leaders for Compassionate Care Programme (LCCP) [69–71], which aims to empower leaders while supporting their teams in delivering high-quality and compassionate patient-centred care [69, 70]. These studies varied in design – one being a quantitative cross-sectional survey [69], and two being qualitative and mixed-methods longitudinal designs [70, 71]. Two studies explored the impact of the LCCP on participants’ personal development, learning experience, service and care delivery, and overall satisfaction with the program; one of these studies identified factors that can embed compassionate care in healthcare environments [69, 70]. In one study, there were reported improvements in participants’ perceived ability to show respect and empathy in their interactions with patients [69]. The study authors also reported that the program was felt to increase motivation and confidence in leading the delivery of compassionate care [69]. A conceptual model was offered by MacArthur et al. (2017), centering on ‘compassionate care’ where the needs of patients, relatives and staff are viewed as being distinct, and on the other hand, inter-related, in which sustainability requires a focus on relationship-centred care mediated through relational practice and relational inquiry, and a need for investment in infrastructure and leadership at both the strategic and local levels [70].

The LCCP also influenced ways of working and specific practice development techniques – particularly, staff receiving regular feedback from patients on how their delivery of compassion influenced their communication with their patients [70]. Smith et al. (2017) evaluated how the LCCP impacted participants’ ability to listen, learn, and respond to patient feedback – a practice that reportedly improved compassion [71], with staff finding value in the experiences of sharing and learning from feedback.

## Discussion

### State of the science: the ongoing monotony, persistent gaps, and incremental progress of compassion research in healthcare

This scoping review provides an updated synthesis of the current literature on the topic of compassion in healthcare over the past 5 years (2015-2020), in keeping with the methodology of the original scoping review that was conducted by members of the Compassion Research Lab [1]. Since the publication of the original scoping review, studies presenting exclusively on patient conceptualizations of compassion have increased (nine studies in total compared to only two that were identified previously), addressing a previously identified limitation—the underrepresentation of the recipients of compassion – patients themselves. This updated review also revealed that HCP and patient perspectives on compassion and compassionate behaviours traversed the themes and subthemes that were previously identified (Table 1), including but not limited to temporal aspects of compassion (i.e. situational in nature, with an ebb and a flow), interpersonal features (i.e. relational care and clinical communication), action, and practicality. While HCPs and patients also identified numerous barriers and enablers to compassion, adaptive behaviours to overcome challenges to compassion were reported in numerous studies coinciding with a general aversion by participants – the notion of absolute barriers to dynamic nature and robustness of compassion. This suggests that in relation to compassion, barriers need to be reconceptualized as challenges—challenges that can be overcome.

### The nature and conflation of compassion: the need for conceptual specificity

In regards to the nature of compassion, while a lack of conceptual specificity persists, additional research focused on the construct of compassion in healthcare over the last 5 years, including the establishment of empirical models of compassion, has produced a growing consensus that compassion is inherently relational, consisting of acknowledging, engaging and proactively attending to another person’s suffering that stems from the innate qualities and good intentions of a fellow human being [6, 45, 79, 82, 87, 88, 101]. The centrality and willingness to proactively address multifactorial suffering, is not only the central aim of palliative care [40–42], but is a defining feature of a compassionate relationship in comparison to other forms of relationships, including empathetic and caring relationships [44]. HCP participants in multiple studies were clear that compassion was other-orientated, was predicated in suffering, and required action aimed at alleviating it [6, 82, 87]. While conceptual clarity and consensus has grown since our original study, additional research over this period also identified some slight cultural variances in relation to compassion, specifically in how it is both expressed and experienced. For example, while there were similarities in how Greek and Cyprus participants perceived compassion, differences also persisted in their definitions, with more than half of the Cyprus participants defining compassion as “empathy and kindness”, whereas Greek participants were more likely to define it as “a deep awareness of the suffering of others and a wish to alleviate it” [88]. However, caution should be exercised in attributing these results strictly to ‘cultural difference’, as is evident in further interpretation of these study results that one plausible reason for these differences is the fact that the Greek participants were practicing registered nurses, whereas those from Cyprus were nursing students who had less clinical experience and exposure to patient suffering. Further, although patients have clearly delineated compassion from empathy and sympathy [44], a couple of included studies utilized definitions of compassion that embedded the term or aspects of empathy [77, 103]. Despite established differences between these terms, one study argued that compassion and empathy are in fact interdependent [98], while participants in another study, concluded that empathy was subsumed within compassion, with compassion enhancing components of empathy while adding action [95]. Despite this lack of conceptual clarity, the attributes or skills comprising compassion were recognized across most of the studies, including the dynamic, responsive, and proactive nature of compassionate action, in comparison to a more static, reactionary, and passive nature of empathy, sympathy, and routine care [44, 50, 86, 92, 99, 107].

### Clinical and educational compassion interventions: can compassion be taught?

The notion of whether one can be trained to become compassionate remains the topic of ongoing debate within the literature, although this debate has dissipated since the previous review. Antecedents, in the form of inherent virtues or personal qualities, and previous personal experiences of suffering and receiving compassion have been previously identified as facilitators of compassion [1, 6, 45, 101, 110, 111]. Studies within this current review have extended what previously was a largely dichotomous (nature vs. nurture) approach to this issue to a more intersectional understanding, comprised of various factors [80, 94]. This complex relationship between intrapersonal factors embedded within individual HCPs and interpersonal factors embedded in the relational and clinical space, was advanced by Uygur in their Compassion Trichotomy [94], which highlights the importance and interdependence of motivation (personal reflection and values), capacity (awareness and regulation of energy, emotion, and cognition), and connection (sustained patient–physician relationship) which influences physicians’ level of compassion [94]. Other studies also highlighted intrinsic altruistic motivators (e.g. personal attitudes, virtues) as catalysts, but not preconditions to providing or enhancing compassion [45, 76, 80, 98, 106]. While there is ongoing debate on whether virtues themselves can be cultivated, we have reported elsewhere that virtues can be cultivated, however the outcomes of this training will vary based on the innate virtues that trainees possess at baseline [50].

### Clinical and educational compassion interventions: how do we teach compassion?

An equally compelling question related to compassion training, arising from studies in this updated review, is how and what are the best methods for cultivating compassion amongst practicing clinicians. While studies suggested that compassion could be cultivated [82, 88, 95] and broad educational approaches such as personal development practices were proposed [95], the intricacies of how and what would be required in a training program remained largely unexplored. While participants in other studies, provided suggestions for teaching methods associated with compassion training [50, 94, 101, 106], including the use of compassion-inducing imagery, sharing heartfelt stories or narratives [75, 93], being exposed to compassionate role models and leadership [50, 106], and through using an experiential approach to learning involving mentorship and self-reflection [50, 79, 101], these recommendations lacked augmentation with educational studies investigating these issues specifically. While one study aimed to investigate the impact of common humanity scenarios on cultivating compassion [75], results from this study were largely predicated on the relational features of compassion and failed to include its action component [75]. Although this study and others focus exclusively on enhancing elements of affective compassion in HCPs [75, 93], viewing common humanity scenarios and interventions focused on perspective-taking of HCPs towards patients, fails to address the multiple domains that comprise compassion and the potential benefit of interventions aimed at enhancing patient perspective-taking towards HCPs [6, 45, 61]. Regardless of these shortcomings, the need to develop, enhance, and sustain a culture of compassion in complex healthcare systems is well-recognized [24–28, 113, 114]. A recent realist review [54] and environmental scan [52] on compassion education literature revealed the intricacies of compassion education programs, describing what works for whom and in what context, which could ultimately inform the development of a comprehensive, evidence-based, clinically-informed compassion training program for HCPs. An imperative, and neglected, factor to cultivating and sustaining compassion in healthcare identified in this recent realist review, was the role of healthcare system and organizational leaders in creating the conditions, educational resources, and policies to ensure that compassion is not only embedded across the healthcare system, but is considered a shared responsibility, and not simply the onus of HCPs [54]. A recent systematic review of predictors of physician compassion revealed similar findings, namely that research on the barriers and facilitators to compassion in healthcare remains disproportionately practitioner-centric, requiring greater research on the both the patient perspective and the influence of broader organizational and system factors [61].

### Clinical and educational compassion interventions: can we measure compassion?

Surprisingly in this updated review, only four of the studies pertained to evaluating compassion educational or clinical interventions – a notable decrease from the 10 interventions identified in the previous review [1]. While one intervention study’s primary aim was to evaluate the Creating Learning Environments for Compassionate Care (CLECC), an educational programme focused on developing managerial and team practices at a group level to enhance team capacity to provide compassionate care for patients (Table 4), researchers utilized the Jefferson Scale of Empathy (JSE) to obtain a nurse-reported measure of empathy at baseline and follow-up [97], rather than using a valid and reliable measure of compassion—the construct of interest. It is interesting to note that the rational for using the JSE in this intervention study was attributed to the fact that the authors were unable to identify a sufficiently psychometrically robust, valid, and reliable measure for compassion, affirming the findings of a previous systematic review of existing compassion measures [55]. The lack of a sufficiently robust compassion measure in this and other intervention studies has been a significant impediment in the advancement of the field and the validity of these compassion interventions, further conflating the concepts of compassion and empathy in the process. The inherent limitations of previous compassion measures were recently addressed in the development of the Sinclair Compassion Questionnaire (SCQ) [15, 55] – a psychometrically rigorous and robust patient-reported compassion measure. The other three intervention studies identified were educational interventions conducted in the UK, aimed towards HCPs, which analyzed the Leaders for Compassionate Care Program (LCCP) within the hospital settings, none of which included patient outcomes [69–71]. Additionally, while the results of these studies focused heavily on participants’ overall satisfaction with the LCCP programme itself, whether it actually improved compassion to patients and families was precariously not assessed.

### Challenges and enablers to compassion

With respect to the literature on challenges and enablers of compassion within practice settings, time constraints, workloads, and staff shortages, remained a prevalent issue in this updated review, as was the case in our original review [72–74, 77, 79, 81, 84, 92, 94, 95, 100, 101, 106, 112]. Despite this persistent challenge, both HCP and patient participants felt that forging a compassionate connection between patients and HCPs could be established in the moment, through ones’ demeanor, the tenor of care, intention, and presence within even the shortest of interactions [6, 45, 73].

### Limitations

There are a few limitations to this updated review**.** First**,** despite applying a robust methodology to identify eligible studies, it is possible that relevant studies could have been inadvertently missed. Secondly, since only English publications were included, we recognize that numerous non-English studies on compassion were excluded, thus limiting generalizability to other non-English speaking settings. Thirdly, in utilizing the previously identified thematic framework generated from the original review in synthesizing the studies within this current review, there is a possibility that this hindered the emergence of additional themes. This decision was purposeful on the part of the review authors in order to remain methodologically congruent with our original review, a decision that we nonetheless were cognisant of in allowing new categories to emerge from the results through a consensus process, thereby avoiding the force fitting of current studies into a predetermined framework. Fourth, while the evidence that self-compassion improves compassion is lacking [115], in excluding intervention studies that focused on improving self-compassion as a means to create more compassionate HCPs, there is a remote possibility that pertinent results were missed. Lastly, despite their inclusion in the original study, studies focusing solely on medical students, trainees, or residents were excluded (except when combined with HCP participants), for the sake of feasibility and because our primary focus was practicing HCPs– who are frequently exposed to patient suffering in a healthcare system were compassion is challenged.

### Implications

An empirically-based, clinically-relevant, patient orientated definition of compassion, that reflects the dynamic nature and multiple domains of the construct of interest is imperative to the fidelity and advancement of educational and clinical interventions designed to improve it. In our original review, there was a notable paucity of studies that conceptualized compassion from not only the perspectives of those who receive it, but also those who strive to provide it—where compassion and suffering reside [1]. Since our original review, targeted efforts to establish the conceptual foundation of compassion were undertaken in various studies identified herein, including but not limited to the development of models of compassion from the perspectives of both patients and clinicians alike. These models of compassion further provide an empirical blueprint depicting the nature, components, flow, facilitators, and inhibitors of compassion for use in research, education, and practice. While these recent studies addressed a conceptual gap identified in the original review, a growing theory-practice gap has emerged in its place over the last 5 years between researchers and HCPs’ knowledge of compassion and their ability to adequately assess it in research and address in clinical practice. While the recent development of a psychometrically rigorous and robust patient-reported compassion measure has partially addressed this issue, there is now a critical need to further address this theory-practice gap through the development of evidence-based educational training programs that equip practicing HCP with the attitudes, knowledge, skills, and behaviours that comprehensively traverse each of the domains of compassion. Similarly, there is a pressing need for RCTs, including future 3-arm RCTs that compare the compassion intervention group to not only standard care, but other related educational interventions such as empathy training. Furthermore, since cultural and gender differences pertaining to how compassion is both expressed and experienced were alluded to within the studies reviewed herein, these individuals and differences must evolve from the realm of platitudes and good intentions to the realm of research priorities and action. While compassion was affirmed as a universal concept in this review, compassion also seeks to understand the uniqueness of the person and their individual needs—whether those individuals are patients or practicing HCPs. Future studies on the topic of compassion need to investigate and honour these differences, whether in the form of validating existing definitions, measures, and interventions of compassion within various cultures, genders, or individuals who experience systemic inequities in care and in society more broadly. Lastly, while assessing the transferability of recently developed valid and reliable patient compassion measures is needed, the existence and further development of valid and reliable research tools offers the ability to begin to meaningfully assess these differences, and provides the means to assess and deliver personalized compassion.

## Conclusion

Since the publication of original scoping review 6 years ago, research on the topic of compassion in healthcare while seeing considerable advances, remains largely theoretical in nature, with limited educational and clinical intervention studies. Despite these limitations, compassion has received increasing attention from researchers, policy makers, educators, HCPs, and particularly patients who consistently identify compassion as a central feature of their overall experience of healthcare. With a firm conceptual foundation of compassion now established with the perspectives of patients embedded therein, greater attention needs to focus on addressing the growing theory-practice gap between what is empirically known and implemented into training and practice. Additional research is needed on developing compassion training programs that honour and are tailored to individuals—including but not limited to their gender identity and cultural background.

## Supplementary Information

**Additional file 1.** Search strategy

## Data Availability

The datasets generated and/or analysed during the current study are available from the corresponding author on reasonable request.

## Abbreviations

HCPs: Healthcare Providers
RCTs: Randomized Controlled Trials
CLECC: Creating Learning Environments for Compassionate Care
LCCP: Leaders for Compassionate Care Program
