# Supplementary material for: Compassion in healthcare: an updated scoping review of the literature
Source: BMC Palliat Care. 2022 May 18;21:80. doi: 10.1186/s12904-022-00942-3 (PMC9116004; doi:10.1186/s12904-022-00942-3)
Supplement: Supplementary file 1 — Additional file 1. Search strategy [file 12904_2022_942_MOESM1_ESM.pdf]

Additional file 1: Search Strategy

Compassion in Healthcare: An Updated Scoping Review of the Literature
November 2020

Database(s): Ovid MEDLINE(R) and Epub Ahead of Print, In-Process & Other Non-Indexed Citations and Daily 1946 to November 16, 2020
Search Strategy:

#	Searches	Results
1	compassion.tw,kf.	6249
2	((compassionate or empathetic) adj1 (care or caring or communication or response* or practice* or engagement)).tw,kf.	1321
3	or/1-2	7260
4	professional-patient relations/ or nurse-patient relations/ or Physician-Patient Relations/	134238
5	interpersonal relations/ or professional-family relations/	87558
6	"Attitude of Health Personnel"/	123187
7	Patient-Centered Care/ or Patient satisfaction/	100665
8	patients/ or inpatients/ or Outpatients/	57401
9	faculty, medical/ or faculty, nursing/ or medical staff/ or nursing staff/	46634
10	Nursing Staff, Hospital/ or Medical Staff, Hospital/	65781
11	"Internship and Residency"/	50554
12	education, nursing/ or education, nursing, baccalaureate/	50731
13	education, medical/ or education, medical, graduate/ or education, medical, undergraduate/	107350
14	students, health occupations/ or students, medical/ or Students, Nursing/	61528
15	exp Nurses/ or exp Physicians/ or exp Health Personnel/ or Caregivers/	523250
16	((relationship-centered or relationship-centred or patient-centered or patient-centred) adj2 (care or caring or approach* or interaction*)).tw,kf.	10738
17	(patient* or nurse* or physician* or practitioner* or clinician* or therapist* or caregiver*).tw,kf.	7381382
18	(resident* or student*).tw,kf.	468113
19	(faculty or professor* or lecturer* or instructor*).tw,kf.	76983
20	or/4-19	8209142
21	3 and 20	5088
22	limit 21 to yr="2015 -Current"	2622
23	limit 22 to english language	2549

Medline
November 27, 2020
Database(s): **Embase** 1974 to 2020 November 25
Search Strategy:

#	Searches	Results
1	compassion.tw,kw.	7618
2	((compassionate or empathetic) adj1 (care or caring or communication or response* or practice* or engagement)).tw,kw.	1644
3	or/1-2	8904
4	professional-patient relationship/ or doctor patient relationship/ or nurse patient relationship/	47384
5	human relation/	88520
6	health personnel attitude/ or nurse attitude/ or physician attitude/	167775
7	patient satisfaction/ or patient attitude/	206669
8	patient/ or hospital patient/ or outpatient/	1672419
9	medical staff/ or hospital personnel/ or medical personnel/ or nursing staff/	139260
10	medical education/ or residency education/ or postgraduate education/	252478
11	nursing education/	82975
12	medical student/ or health student/	72399
13	nursing student/	26125
14	exp nurse/ or exp physician/ or caregiver/	989652
15	((relationship-centered or relationship-centred or patient-centered or patient-centred) adj2 (care or caring or approach* or interaction*)).tw,kw.	14648
16	(patient* or nurse* or physician* or practitioner* or clinician* or therapist* or caregiver*).tw,kw.	10578285
17	(resident* or student*).tw,kw.	613613
18	(faculty or professor* or lecturer* or instructor*).tw,kw.	98899
19	or/4-18	11657350
20	3 and 19	6426
21	limit 20 to yr="2015 -Current"	3382
22	limit 21 to conference abstracts	833
23	21 not 22	2549
24	limit 23 to english language	2480

CENTRAL
November 27, 2020

Database(s): **EBM Reviews - Cochrane Central Register of Controlled Trials** October 2020
Search Strategy:

#	Searches	Results
1	compassion.tw,kw.	860
2	((compassionate or empathetic) adj1 (care or caring or communication or response* or practice* or engagement)).tw,kw.	61
3	or/1-2	908
4	professional-patient relations/ or nurse-patient relations/ or Physician-Patient Relations/	2538
5	interpersonal relations/ or professional-family relations/	2439
6	"Attitude of Health Personnel"/	1979
7	Patient-Centered Care/ or Patient satisfaction/	11986
8	patients/ or inpatients/ or Outpatients/	2442
9	faculty, medical/ or faculty, nursing/ or medical staff/ or nursing staff/	338
10	Nursing Staff, Hospital/ or Medical Staff, Hospital/	688
11	"Internship and Residency"/	1266
12	education, nursing/ or education, nursing, baccalaureate/	497
13	education, medical/ or education, medical, graduate/ or education, medical, undergraduate/	1633
14	students, health occupations/ or students, medical/ or Students, Nursing/	1500
15	exp Nurses/ or exp Physicians/ or exp Health Personnel/ or Caregivers/	8578
16	((relationship-centered or relationship-centred or patient-centered or patient-centred) adj2 (care or caring or approach* or interaction*)).tw,kw.	1007
17	(patient* or nurse* or physician* or practitioner* or clinician* or therapist* or caregiver*).tw,kw.	1035570
18	(resident* or student*).tw,kw.	49504
19	(faculty or professor* or lecturer* or instructor*).tw,kw.	5430
20	or/4-19	1070500
21	3 and 20	585
22	limit 21 to yr="2015 -Current"	483
23	limit 22 to english language	277

PsycINFO
November 27, 2020

Database(s): **APA PsycInfo** 1806 to November Week 3 2020
Search Strategy:

#	Searches	Results
1	compassion.tw,id.	8888
2	((compassionate or empathetic) adj1 (care or caring or communication or response* or practice* or engagement)).tw,id.	795
3	or/1-2	9454
4	Therapeutic Processes/ or interpersonal relationships/	44576
5	health personnel attitudes/	19931
6	patient centered care/	233
7	client satisfaction/	5645
8	patients/ or hospitalized patients/ or outpatients/	45234
9	medical education/ or medical internship/ or medical residency/ or nursing education/	27681
10	medical students/ or nursing students/	18639
11	exp nurses/ or exp medical personnel/ or exp physicians/	83031
12	exp nurses/ or exp medical personnel/ or exp physicians/ or exp caregivers/	111325
13	((relationship-centered or relationship-centred or patient-centered or patient-centred) adj2 (care or caring or approach* or interaction*)).tw,id.	3152
14	(patient* or nurse* or physician* or practitioner* or clinician* or therapist* or caregiver*).tw,id.	992561
15	(resident* or student*).tw,id.	664918
16	(faculty or professor* or lecturer* or instructor*).tw,id.	66225
17	or/4-16	1658085
18	3 and 17	4712
19	limit 18 to yr="2015 -Current"	2276
19	limit 20 to peer reviewed journal	1487

CINAHL
November 27, 2020

#	Query	Results
S1	TI compassion OR AB compassion	5,765
S2	TI (((compassionate or empathetic) N1 (care or caring or communication or response* or practice* or engagement))) OR AB (((compassionate or empathetic) N1 (care or caring or communication or response* or practice* or engagement)))	1,569
S3	S1 OR S2	7,024
S4	(MH "Interpersonal Relations") OR (MH "Interprofessional Relations") OR (MH "Intraprofessional Relations") OR (MH "Patient-Family Relations") OR (MH "Nurse-Physician Relations") OR (MH "Professional-Client Relations") OR (MH "Professional-Family Relations") OR (MH "Professional-Patient Relations") OR (MH "Nurse-Patient Relations") OR (MH "Physician-Patient Relations")	203,826
S5	(MH "Attitude of Health Personnel") OR (MH "Nurse Attitudes") OR (MH "Physician Attitudes")	90,322
S6	(MH "Patient Centered Care")	31,020
S7	(MH "Patient Satisfaction")	56,112
S8	(MH "Patients") OR (MH "Outpatients") OR (MH "Inpatients")	134,246
S9	(MH "Faculty, Medical") OR (MH "Faculty, Nursing") OR (MH "Medical Staff") OR (MH "Medical Staff, Hospital") OR (MH "Nursing Staff, Hospital")	46,659
S10	(MH "Internship and Residency")	17,609
S11	(MH "Education, Nursing") OR (MH "Education, Nursing, Baccalaureate") OR (MH "Education, Nursing, Graduate")	61,001
S12	(MH "Education, Medical") OR (MH "Education, Health Sciences")	32,908
S13	(MH "Students, Health Occupations") OR (MH "Students, Allied Health") OR (MH "Students, Medical") OR (MH "Students, Nursing") OR (MH "Students, Nursing, Baccalaureate") OR (MH "Students, Nursing, Graduate")	55,005
S14	(MH "Nurses+") OR (MH "Physicians+") OR (MH "Health Personnel+") OR (MH "Allied Health Personnel+") OR (MH "Caregivers")	619,824
S15	TI (((relationship-centered or relationship-centred or patient-centered or patient-centred) N2 (care or caring or approach* or interaction*))) OR AB (((relationship-centered or relationship-centred or patient-centered or patient-centred) N2 (care or caring or approach* or interaction*)))	7,144
S16	TI ((patient* or nurse* or physician* or practitioner* or clinician* or therapist* or caregiver*)) OR AB ((patient* or nurse* or physician* or practitioner* or clinician* or therapist* or caregiver*))	2,202,624
S17	TI ((resident* or student*)) OR AB ((resident* or student*))	244,191
S18	TI ((faculty or professor* or lecturer* or instructor*)) OR AB ((faculty or professor* or lecturer* or instructor*))	44,033
S19	S4 OR S5 OR S6 OR S7 OR S8 OR S9 OR S10 OR S11 OR S12 OR S13 OR S14 OR S15 OR S16 OR S17 OR S18	2,930,356
S20	S3 AND S19	5,128
S21	S3 AND S19<br>Limiters - Published Date: 20150101-20211231	2,618
S22	S3 AND S19<br>Limiters - Published Date: 20150101-20211231; Scholarly (Peer Reviewed) Journals	2,241
S23	S3 AND S19<br>Limiters - Published Date: 20150101-20211231; Scholarly (Peer Reviewed) Journals<br>Narrow by Language: - english	2,179

Academic Search Complete
November 27, 2020

#	Query	Results
S1	TI compassion OR AB compassion OR KW compassion	12,553
S2	TI ((((compassionate or empathetic) N1 (care or caring or communication or response* or practice* or engagement)))) OR AB ((((compassionate or empathetic) N1 (care or caring or communication or response* or practice* or engagement)))) OR KW ((((compassionate or empathetic) N1 (care or caring or communication or response* or practice* or engagement))))	1,441
S3	S1 OR S2	13,698
S4	DE "INTERPERSONAL relations" OR DE "MEDICAL personnel-caregiver relationships" OR DE "NURSE-patient relationships" OR DE "NURSE-physician relationships" OR DE "PATIENT-family relations" OR DE "PATIENT-professional relations" OR DE "PHYSICIAN-patient relations"	107,823
S5	DE "ATTITUDES of medical personnel" OR DE "PHYSICIANS' attitudes" OR DE "NURSES' attitudes"	12,817
S6	DE "PATIENT-centered care"	11,496
S7	DE "PATIENT satisfaction"	22,939
S8	DE "PATIENTS" OR DE "HOSPITAL patients"	256,707
S9	DE "MEDICAL school faculty" OR DE "HEALTH occupations school faculty" ORDE "NURSING school faculty"	1,247
S10	DE "HOSPITAL medical staff" OR DE "HOSPITAL personnel" OR DE "HOSPITAL nursing staff" OR DE "INTERNS (Medicine)" OR DE "RESIDENTS (Medicine)" ORDE "HOSPITAL nursing staff"	12,360
S11	DE "NURSING education" OR DE "BACCALAUREATE nursing education" OR DE "GRADUATE nursing education" OR DE "MEDICAL education" OR DE "ALLIED health education" OR DE "GRADUATE medical education" OR DE "HEALTH education"	63,546
S12	DE "NURSING students" OR DE "HEALTH occupations students" OR DE "MEDICAL students"	33,754
S13	DE "NURSES" OR DE "PHYSICIANS" OR DE "MEDICAL personnel" OR DE "CAREGIVERS" OR DE "ALLIED health personnel"	194,471
S14	TI ((((relationship-centered or relationship-centred or patient-centered or patient-centred) N2 (care or caring or approach* or interaction*)))) OR AB ((((relationship-centered or relationship-centred or patient-centered or patient-centred) N2 (care or caring or approach* or interaction*)))) OR KW ((((relationship-centered or relationship-centred or patient-centered or patient-centred) N2 (care or caring or approach* or interaction*))))	7,468
S15	TI (((patient* or nurse* or physician* or practitioner* or clinician* or therapist* or caregiver*))) OR AB (((patient* or nurse* or physician* or practitioner* or clinician* or therapist* or caregiver*))) OR KW (((patient* or nurse* or physician* or practitioner* or clinician* or therapist* or caregiver*)))	3,452,564
S16	TI (((resident* or student*))) OR AB (((resident* or student*))) OR KW (((resident* or student*)))	992,295
S17	TI (((faculty or professor* or lecturer* or instructor*))) OR AB (((faculty or professor* or lecturer* or instructor*))) OR KW (((faculty or professor* or lecturer* or instructor*)))	255,487
S18	S4 OR S5 OR S6 OR S7 OR S8 OR S9 OR S10 OR S11 OR S12 OR S13 OR S14 OR S15 OR S16 OR S17	4,698,715
S19	S3 AND S18	5,067
S20	S3 AND S18<br>Limiters - Published Date: 20150101-20211231	2,464
S21	S3 AND S18<br>Limiters - Published Date: 20150101-20211231; Scholarly (Peer Reviewed) Journals	2,201
S22	S3 AND S18<br>Limiters - Published Date: 20150101-20211231; Scholarly (Peer Reviewed) Journals<br>Narrow by Language: - english	2,090

SCOPUS
November 27, 2020
3,104

((TITLE-ABS-KEY (compassion) OR TITLE-ABS-KEY (((compassionate OR empathetic) W/1 (care OR caring OR communication OR response* OR practice* OR engagement))))) AND ((TITLE-ABS-KEY (((relationship-centered OR relationship-centred OR patient-centered OR patient-centred) W/2 (care OR caring OR approach* OR interaction*))) OR TITLE-ABS-KEY ((patient* OR nurse* OR physician* OR practitioner* OR clinician* OR therapist* OR caregiver*)) OR TITLE-ABS-KEY ((resident* OR student*)) OR TITLE-ABS-KEY ((faculty OR professor* OR lecturer* OR instructor*)))) AND (LIMIT-TO (PUBYEAR , 2021) OR LIMIT-TO (PUBYEAR , 2020) OR LIMIT-TO (PUBYEAR , 2019) OR LIMIT-TO (PUBYEAR , 2018) OR LIMIT-TO (PUBYEAR , 2017) OR LIMIT-TO (PUBYEAR , 2016) OR LIMIT-TO (PUBYEAR , 2015)) AND (LIMIT-TO (DOCTYPE , "ar")) AND (LIMIT-TO (LANGUAGE , "English"))

Downloaded files
Scopus 2021,2020, 2019, 2018 = 1936
Scopus 2017, 2016, 2015 = 1168
